# Locomotive Syndrome: Operational Definition Based on a Questionnaire, and Exercise Interventions on Mobility Dysfunction in Elderly People

**DOI:** 10.1007/s12018-016-9210-8

**Published:** 2016-06-03

**Authors:** M Akai, T. Doi, A. Seichi, Y. Okuma, T. Ogata, T. Iwaya

**Affiliations:** Graduate School, International University of Health and Welfare, 4F, Aoyama 1-Chome Tower, 1-3-3 Minami-Aoyama, Minato-ku, Tokyo, 107-0062 Japan; Geriatric Care Facility Hakucho, 3-18-24 Tabata, Kita-ku, Tokyo, 114-0014 Japan; Department of Orthopedics, Mitsui Memorial Hospital, 1 Kanda-Izumi-chou, Chiyoda-ku, Tokyo, 101-0024 Japan; National Rehabilitation Center for Persons with Disabilities, 4-1 Namiki, Tokorozawa, Saitama, 359-8555 Japan; Nagano University of Health and Medicine, 11-1 Imaihara, Kawanakajima-chou, Nagano, 381-2227 Japan

**Keywords:** Aging society, Long-term care, Locomotive disorders, Physical activity, Exercise

## Abstract

The increasing elderly population has a great impact on public health, and it is important to understand the progression of musculoskeletal disorders seen in this population. To establish useful preventative methods for such locomotive disorders, we must detect early changes in these individuals and identify those at risk in order to implement early interventions. The purpose of this review was: (1) to introduce an operational definition of locomotion dysfunction to prevent a care-need condition, and to verify its validity through a prospective cohort study, and (2) to review the indication of exercise intervention for multiple musculoskeletal involvements from the preceding literature. We developed a measurement scale called the Geriatric Locomotive Function Scale (GLFS)-25, which clearly reflects the degree of functional deterioration. We used it in a prospective cohort study of 314 patients recruited from 5 clinics or nursing care facilities and investigated the relationship of the GLFS-25 with 46 variables covering various clinical manifestations. The results clearly revealed that the change in the GLFS-25 classification reflected a common pattern seen in those with locomotive dysfunction. Recently, several important movements regarding physical activity and its public promotion have been advocated by international health organizations and journal publishers. Though it has not been confirmed yet that complex musculoskeletal diseases can be treated using therapeutic exercise, the promotion of physical activity appears promising. The degree of activity limitation in aged individuals with locomotive disorders can be evaluated using this scale, which may be useful in predicting the effectiveness of future interventions.

## Introduction

Increase in the aging population has a great impact on public health, and the proportion of elderly individuals aged ≥65 years in Japan was 26.0 % in 2014 [1]. It is important to understand the progression of disorders in this population because a dramatic increase in the number of elderly requiring help from others is an urgent matter from the medical and socioeconomical points of view [2, 3]. According to an analysis of the long-term care insurance of Japan, the common conditions for which long-term care services are sought by individuals aged over 65 years are cerebrovascular disorders (18.5 %), dementia (15.8 %), and, importantly, problems related to the locomotive organs, such as falls/fractures (11.8 %), joint disorders (10.9 %), and aging frailty (13.4 %) [4]. Furthermore, a rapid increase in those people happens mainly in a group of early or mild stage requiring not care-need but some support without obvious clinical disability.

Locomotive organ disorders are problematic in a super-aged society, as most of these have a chronic course and affect a large number of older persons. Approximately 47 million people have at least 1 of the following 3 disorders: radiographic knee osteoarthritis, radiographic lumbar spondylosis, and osteoporosis which is diagnosed based on an assessment of the bone mineral density. In addition, many individuals experience multiple locomotive organ disorders. An estimated 24.7 million people have 2 of the above-mentioned disorders, and 5.4 million have all 3 [5].

The health of aged individuals is of great concern, not only to themselves but also to the general public. The Japanese Orthopaedic Association (JOA) has proposed the concept of “locomotive syndrome,” which refers to (1) conditions that need nursing care services, or (2) high-risk conditions that will require such services soon because of locomotive organ dysfunction in the elderly [6, 7]. However, we do not have sufficient knowledge to cover such wide range of musculoskeletal diseases that are usually accompanied by pain as the main complaint. We have to establish an approach for such multiple involvements, as the elderly generally have several problems and develop complicated conditions [8, 9].

We have a considerable amount of the literature available on the effectiveness of therapeutic exercise in those with musculoskeletal disorders. However, these diseases are individually diagnosed as a conventional single criterion and managed with therapeutic exercise for targeted organs or symptoms. The effectiveness of exercise intervention on the overall deterioration of motor function as well as disability during performance of daily activities has not been sufficiently investigated in the elderly with multiple involvement of the musculoskeletal system.

The purpose of this article was (1) to introduce our study, which was conducted to establish an operational definition of locomotive dysfunction for preventing the care-need condition and verify the validity of this definition through a prospective cohort study and (2) to review the direction of intervention using therapeutic exercise for multiple musculoskeletal involvement from the existing literature.

According to the assumption that the elderly have complicated multiple involvements, relationships between the signs and symptoms, several measurements in locomotor function, daily activities, and social participation were statistically investigated.

## Methods

### Part 1: Locomotive Disability Prevention (LDP) Study

#### Study Design

We designed a prospective cohort study to investigate locomotive organ dysfunction in older individuals. This study was approved by the ethical committee of the Japanese Orthopaedic Association and supported by a Sciences Research Grant from the Ministry of Health, Labor and Welfare, Japan (H21-Choju-G006). We also developed a self-completed numerical questionnaire called the Geriatric Locomotive Function Scale-25 (GLFS-25) (Table 1), the validity and reliability of which had already been assessed from the psychometric point of view [10].

**Table 1:** Geriatric Locomotive Function Scale 25: Question and answer items

Questions	Selection of answer
1. Any pain (including numbness) in your neck or upper limb (shoulder, arm, or hand)	1. No pain 2. Mild pain 3. Moderate pain 4. Considerable pain 5. Severe pain
2. Any pain in your back, lower back, or buttocks	
3. Any pain (including numbness) in your lower limb (hip, thigh, knee, calf, shin, ankle, or foot)	
4. Painful to move the body in daily life	
5. Difficult to get up from a bed or lie down	1. Not difficult 2. Mildly difficult 3. Moderately difficult 4. Considerably difficult 5. Extremely difficult
6. Difficult to stand up from a chair	
**7.** Difficult to walk inside the house	
8. Difficult to put on and take off shirts	
9. Difficult to put on and take off trousers and pants	
10. Difficult to use the toilet	
11. Difficult to wash the body in the bath	
12. Difficult to go up and down stairs	
13. Difficult to walk briskly	
14. Difficult to keep oneself neat	
15. How far can you walk without resting?	1. More than 2–3 km 2. Approximately 1 km 3. Approximately 300 m 4. Approximately 100 m 5. Approximately 10 m
16. Difficult to go out to visit neighbors	1. Not difficult 2. Mildly difficult 3. Moderately difficult 4. Considerably difficult 5. Extremely difficult
17. Difficult to carry objects weighing approximately 2 kg	
18. Difficult to go out using public transportation	
19. Difficult to do simple tasks and housework (preparing meals, cleaning up, etc.)	
20. Difficult to perform load-bearing tasks and housework (cleaning the yard, carrying heavy bed, etc.)	
21. Difficult to perform sports activities (jogging, swimming, gate ball, dancing, etc.)	
22. Restricted from meeting your friends	1. Not restricted 2. Slightly restricted 3. Restricted about half the time 4. Considerably restricted 5. Gave up all activities
23. Restricted from joining social activities (meeting friends, engaging in activities and hobbies, etc.)	
24. Feel anxious about falls in your house	1. Not restricted 2. Slightly restricted 3. Restricted about half the time 4. Considerably restricted 5. Gave up all activities
25. Feel anxious about being unable to walk in the future	

This is a similar scale which is so-called Rocomo-25 in Japanese (https://locomo-joa.jp/en/index.pdf)

### Participants

We recruited participants aged ≥65 years from five orthopedic clinics or affiliated nursing care facilities. Written informed consent was obtained from each participant.

#### Inclusion Criteria

1. Age ≥65 years (either gender)
2. Any one from the following four sources:
  1. Complaints related to the legs or spine without disability in walking or going out (outpatients at orthopedic clinics).
  2. Complaints related to the legs and spine, and minor disabilities in walking and going out (outpatients at orthopedic clinics).
  3. Some disabilities in walking due to locomotive organ disorders (users of long-term care services).
  4. Complaints related to the upper extremities without disability in walking or going out (outpatients at orthopedic clinics) as comparative group.
3. Ability to answer the GLFS-25 questionnaire without assistance.
4. Consent to radiographic examination of the knees and spine.
5. Consent to examination of serum vitamin D and hyaluronic acid levels.
6. Consent to participate in the following motor function tests: one-leg standing test [11], measurements of grip power and leg extension power [12], 100-step test [13], and trunk forward bending test [14].

#### Exclusion Criteria

1. Inability to stand up from a chair or bed.
2. Disability in walking or locomotion due to neurological disease requiring admission.
3. Severe pulmonary, renal, coronary, or hepatic disease.
4. Mental illness.
5. Past history of stroke within the preceding 6 months.
6. Past history of myocardial infarction within the preceding 6 months.
7. Past history of fracture of a lower extremity within the preceding 6 months.
8. Current treatment for acute trauma.
9. Other reasons detected and determined by the attending physician.

The participants enrolled in the outpatient rehabilitation program at the predescribed 5 orthopedic clinics or nursing care facilities; they were assessed 4 times: at baseline, and 6, 12, and 18 months later. In this study, we mainly used the baseline data.

### Collecting and Synthesis of the Data

We asked each participant about his or her history of falls and fractures, regular medications, diagnoses related to the locomotive organs, comorbidities, use of walking aids, living environment (especially number of family members needed for care), and physiotherapeutic interventions, and requested each participant to complete the GLFS-25 questionnaire.

The attending medical staff examined the patient based on the complaints and the specific painful area (back, buttock, thigh, or knee), determined the posture classification according to the classification proposed by Nakata [15], and recorded the physical findings related to the trunk and lower extremities. They also measured the body height, body weight, range of motion (ROM) of the hip and knee joints, and strength of the iliopsoas, quadriceps, anterior tibialis, and calf muscles, and recorded the results of the motor function tests, including the one-leg standing time, grip power, leg extension power, 100-step test, and trunk forward bending distance. The staff obtained radiographs, including an anteroposterior view of the knee joints in a standing posture and a lateral view of the thoracolumbar spine, and assessed them quantitatively using semiautomated computer-aided diagnosis. The bone density of the wrist or metacarpal bones, the lumbar spine, or the proximal femur was measured using X-ray absorptiometry (DEXA), digital image processing (DIP), or quantitative computed tomography (QCT), and expressed as the percentage of the mean for young adults (YAM). Serum vitamin D and hyaluronic acid levels were also examined.

### Creation of Variables

The 10 fields, 42 items, and original 392 variables assessed in the cohort study and the 46 variables used for the present statistical analyses are shown in Table 2.

**Table 2:** Contents of 10 fields, 42 items, and original 392 variables assessed in the participants in cohort study and the 46 variables used for the present statistical analyses

Items (*N* = 42)		Investigated variables (*N* = 392)		Used variables (*N* = 46)
Basic biological data	8	Gender, age, educational and vocational history, approval for long-term care insurance (if available), body height and weight	7	5
Living environment	2	Family structure, condition of house	8	1
Health status	5	Cognitions, mood, visual and auditory problems, need for walking aids	25	8
Medical history and comorbidity	3	Past medical history and comorbidities, medication history	42	3
Locomotive organ problems	4	Complaints, diagnosis for locomotive organ diseases, method of treatment, medical history including fractures or falls	65	3
Physical findings	4	Area of pain (back pain, buttock pain, thigh pain, knee pain), posture classification, neurological signs	19	8
Laboratory tests	5	Serum vitamin D, hyaluronic acid, bone mineral density	3	3
X-rays	2	Semiquantitative X-ray findings related to the spine and knee	159	2
Motor functional assessment	8	Muscle strength, range of motion of hip and knee joints, one-leg standing time, 100-step test, grip strength, lower limb extension power, forward bending, grades of independence	39	12
GLFS-25 score	1	25 items	25	1

Those variables are investigated in the ongoing cohort study, and the results of motor function tests, three laboratory data, and two X-rays relating variables are analyzed in the present study

#### Muscle Strength

Physical therapists evaluated the bilateral iliopsoas, quadriceps, anterior tibialis, and calf muscle strength using manual muscle testing.

If a muscle scored 5 on both sides, its muscle strength was classified as normal; otherwise, it was classified as weak.

#### ROM of the Hip and Knee Joints

Physiotherapists measured the ROM of the hip and knee joints in the supine position.

If the bilateral hip joints exhibited ≥100° of flexion and no limitation of extension, the ROM of the hip joints was classified as normal; otherwise, it was classified as limited.

If the bilateral knee joints exhibited ≥135° of flexion, the ROM of the knee joints was classified as normal; otherwise, it was classified as limited.

#### Pain

The attending physicians examined the lower back, buttocks, bilateral posterior thigh region, and knee joints regardless of whether an individual reported soreness, tenderness, or pain during motion in those areas.

The presence (+) or absence (−) of pain in the lower back was recorded. Similarly, the presence (+) or absence (−) of pain in the buttocks, posterior thigh, or knee joints was also recorded.

#### Neurological Signs

The attending physicians examined the patellar tendon reflex (PTR), Achilles tendon reflex (ATR), and sensory changes in the lower leg bilaterally.

If the PTR was normal bilaterally, the PTR was classified as normal; if the PTR was exaggerated on one or both sides, the PTR was classified as increased; otherwise, the PTR was classified as decreased.

If the ATR was normal, the ATR was classified as normal; otherwise, it was classified as decreased (no participant exhibited an increased ATR).

If the sensory function was normal in both lower legs, the sensory change was classified as “−” and otherwise as “+.”

#### Physical Function

The one-leg standing test measures the time for which the subject can stand on one leg with their eyes open. The one-leg standing time was measured bilaterally, and the mean of 2 trials was calculated.

The grip power was measured bilaterally using a dynamometer, and the stronger value of the 2 was designated as the grip power.

The leg extension power was measured bilaterally using a previously described device [12]. The mean values of both sides were calculated, and the stronger value from the 2 trials was designated as the leg extension power.

The 100-step test measures the time required to step in place 100 times. The shorter value from the 2 trials was designated as the 100-step time.

The trunk-bending test measures the distance between the fingertip and foot sole as the subject bends forward as deeply as possible in a long sitting posture. The longer distance from the 2 trials was designated as the trunk-bending distance.

#### Radiographic Measurements

The radiographs of the knee joints and thoracolumbar spine were examined using specialized computer programs.

Specialized software—the knee osteoarthritis computer-aided diagnosis (KOACAD) program [16]—was used to measure medial and lateral joint space narrowing, osteophyte formation, and joint angulation, i.e., femorotibial angle (FTA, X-ray variables representing knee alignment). This method measures all parameters on plain knee radiographs in a fully automated manner. The medial and lateral joint space areas were measured on both sides and the narrowest joint space area value designated as the minimum joint space area.

On the lateral-view spine radiograph, the vertebral alignment between L1 and S1, vertebral deformity, osteophyte size, intervertebral disk space area, and ratio of sliding were measured using another specialized computer program. Specifically, the lumbosacral angle (LSA, the angle between lines parallel to the upper border of L1 body and sacrum) represents spinal deformity due to degenerative changes in intervertebral disks or the spinal body.

#### Laboratory Findings

Bone density was measured by either DEXA or DIP. The ratio of the bone density to the mean density in young adults was calculated and designated as the YAM. The serum vitamin D and hyaluronic acid were checked.

We used those 46 variables for statistical analyses (Table 2).

### Statistical Handling

Histograms of self-filled questionnaire scores like the GLFS-25 usually show a skewed distribution shifted to the left, like the F-distribution. The test for normality in this study revealed the non-normal distribution of the scores.

We arranged the GLFS-25 scores in ordinal levels (GLFS grade) using “R language” program for optimal classification of histogram [17, 18] and designated the levels according to the degree of disability in activities of daily living (ADL). The ‘R’ is a free software programming language for statistical computing and graphics, and it is widely used among statisticians and data miners for statistical software development and data analysis. We used such software in order to stratify the entire GLFS-25 data mathematically and not arbitrarily.

We examined relationships between variables and degree of severity in ADL disability with Kruskal–Wallis test using GLFS grade.

These variables were checked independently as single correlation with the GLFS-25. As it was not possible to exclude the potential confounding among the variables, we used the Akaike Information criterion (AIC) [19] for its calculation at this stage. AIC, a statistical method, compares the amount of “fitting” between each item combination, and the AIC value represents the amount of association between two items without latent confounding effects [20]. A negative AIC value indicates a stronger association between two items.

The Institute of Statistical Mathematics in Japan developed a computer program named Categorical Data Analysis Program (CATDAP-02) to conduct cross-table analyses with all combinations of questionnaire items [21]. This program simultaneously searches for the best subset and the best categorization of explanatory variables (items), and automatically sorts out good fit combinations using the AIC. We analyzed the relationships between the GLFS-25 and the other 45 variables using the AIC test to identify the variables strongly related to the GLFS-25. AIC values were calculated in all the double and triple combinations among examined data as a round robin to investigate their relationship.

All statistical analyses were performed using the SPSS^®^ version 20 software (SPSS, Chicago, IL, USA).

### Part 2: Literature Review on Physical Activity

#### World Trend on Physical Activity

Recently, several important movements regarding physical activity or effectiveness of exercise and its public promotion have been advocated by the following international health organizations and journal publishers:

1. Toronto Charter for Physical Activity 2010 [22]
2. Global Recommendations on Physical Activity for Health. 2010 [23]
3. Series articles on Physical Activity in The Lancet 2012 [24–27]

We briefly summarized the content of these movements.

### Exercise for Musculoskeletal Disorders: Guidelines and Meta-analysis

Great amount of publication on the effect of exercise for each orthopedic disease has been published. When sufficient evidence has been accumulated to show the significant benefits of a certain intervention, further study cannot overturn such results no more.

However, for a relatively wide disease group such as musculoskeletal diseases, only a few meta-analyses, systematic reviews, and guidelines are available. We investigated the meta-analyses and guidelines on the effectiveness of therapeutic exercise in musculoskeletal conditions, which covered a wide range of diseases. An important aspect is the identification of participants and outcome measures.

## Results

### Characteristics of the Participants

We recruited 314 participants (80 men and 234 women) whose ages ranged from 65 to 93 years. The mean age was 75.9 years (SD 6.3) in men and 77.9 years (SD 8.0) in women. The mean body height and weight in men were 160 cm (SD 6.7) and 60.6 kg (SD 9.8), and in women were 147.0 cm (SD 5.8) and 50.7 kg (SD 8.8), respectively. The number of participants living alone and with 1, 2, and 3 or more family members was 67, 104, 44, and 95, respectively.

The diagnoses for these participants were as follows: knee osteoarthritis (136), osteoporosis (67), spinal canal stenosis (58), spinal spondylitis (54), and multiple diagnoses (133). Numbers of diagnosis per participants were 1 in 161, 2 in 85, and more than 3 in 47. Among this participant group, 268 had comorbidity such as hypertension or diabetes.

A total of 143 participants used walking aids such as a cane or walker. A history of falls was reported by 233 participants, and 158 had a history of fracture within the past few years.

Hip joint ROM was limited in 52 participants and knee joint ROM in 102 participants.

The muscle strength of the iliopsoas muscle was rated as weak in 220 participants.

The muscle strength of the quadriceps was rated as weak in 157 participants.

The muscle strength of the anterior tibialis was rated as weak in 87 participants.

The muscle strength of the triceps surae was rated as weak in 174 participants.

Sensory changes in the lower leg were observed in 32 participants.

The number of participants with low back pain was 212, with gluteal pain was 94, with sciatic pain was 44, and with knee joint pain was 203.

The mean grip power was 20.8 kg (SD 6.9), mean 100-step time was 58.2 s (SD 11.8), mean leg extension power was 51.6 kg (SD 29.5), mean one-leg standing time was 17.5 s (SD 19.6), and mean trunk-bending distance was 27.0 cm (SD 15.8).

The mean serum hyaluronic acid was 123.1 ng/ml (SD 91.6), mean serum 25OH vitamin D was 44.7 ng/ml (SD 22.2), mean bone mineral density was 78.2 % YAM (SD 17.8), mean FTA was 179 (SD 4.7), and mean L1/S was 30.9 (SD 19).

The level of limitations in daily life reflected the degree of support or care-need, as assessed by the attending physicians. Accordingly, a strong relationship was observed between the GLFS-25 questionnaire completed by the participants and their functional status (Kruskal–Wallis test, Monte Carlo sig. *p* < 0.001) (Table 3).

**Table 3:** Functional status of the participants according to the level of limitation of daily life reflected the degree of support or care-need and the GLFS-25 score

		Grading according to long-term care system						Net
		No symptoms in the locomotor system and no limitation in daily life	Symptoms in the locomotor system but no limitation in walking or going out	Symptoms in the locomotor system and some limitation in walking but lives without any assistance	Requires slight assistance due to impaired mobility, but requires no assistance for basic ADL		Requires slight assistance for basic ADL	
					Support 1	Support 2	Care 1 (or more)	
GLFS-25 Score	6	2	25	2	5	1	0	35
	7–15	1	45	16	22	2	1	87
	16–23	0	25	11	25	7	0	68
	24–32	0	9	13	11	6	0	39
	33–40	0	4	10	10	7	0	31
	41–49	0	2	6	11	7	0	26
	50–	0	2	4	7	8	1	22
Cases		3	112	62	91	38	2	308

The GLFS-25 scores were mathematically stratified into seven categories using “R language” program for optimal classification of histogram. Functional status of the participants according to the level of limitation of daily life reflected the degree of support or care-need and the GLFS-25 score (Kruskal–Wallis test, Monte Carlo sig. *p* = 0.000, 99 % Confidence Interval; lower 0.000, upper 0.000)

Instrumental ADL includes household tasks, using a telephone, and taking medicine. Basic ADL includes living indoors, standing up, walking indoors, and taking a bath

Among such extracted variables, 24 variables showed a significant relationship with the GLFS-25, which included comorbidity, mood, and others. The degree of good fitness among the combinations of GLFS-25 and other variables were then examined using AIC, which revealed each association between them.

### Variables Strongly Associated with the GLFS-25

In 15,226 sets of double or triple combination from the 46 used variables (_2_C_46_ and _3_C_46_), 11 variables proved to be strongly associated with the GLFS-25 because of their negative AIC values. Six of these 11 variables were related to signs and symptoms related to the locomotor system: muscle weakness of the triceps surae, presence of sensory impairment in the lower extremity, anterior tibialis weakness, presence of knee pain, presence of low back pain, and quadriceps femoris weakness (Table 4).

**Table 4:** Variables that show negative Akaike information criterion (AIC) values in relation to the GLFS-25 score

Variables investigated in a cohort study	AIC values
Use of walking aids	−18.99
Bothersome to do daily tasks	−15.62
Weakness of triceps surae muscle	−14.83
Sensory change in lower leg	−13.23
Muscle weakness of anterior tibial muscle	−9.18
Difficulty seeing	−7.58
Knee joint pain	−3.53
Difficulty hearing	−2.28
Low back pain	−2.04
Sense of powerlessness (feel oneself disabled)	−2.02
Weakness of quadriceps muscle	−1.96

Among 11 variables which are confirmed by AIC calculation in relation to the GLFS-25 score, following 6 are associated with locomotive signs and symptoms: weakness of calf muscle, sensory change in lower leg, weakness of anterior tibial muscle, knee joint pain, lower back pain, and weakness of quadriceps muscle

**Fig. 1:**
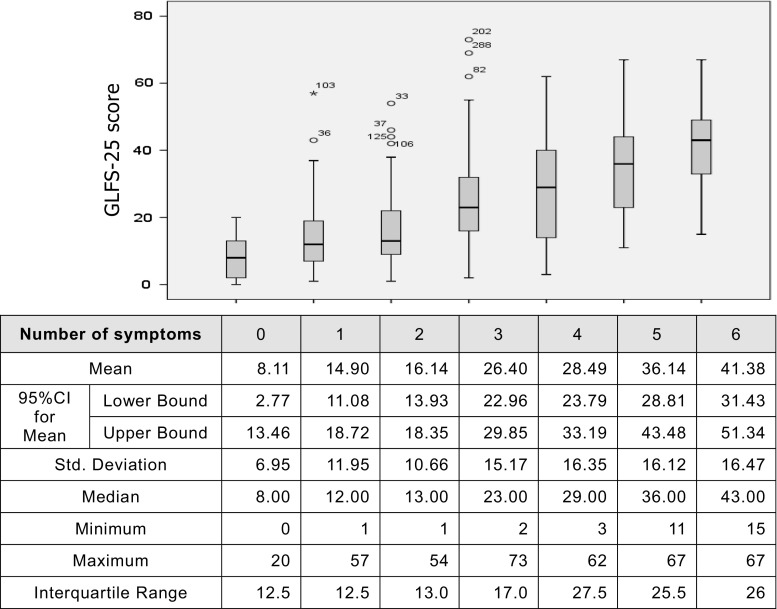
Number of those symptoms relating to locomotor functions revealed significant relationship with the GLFS-25 scores (Kruskal–Wallis test)

**Fig. 2:**
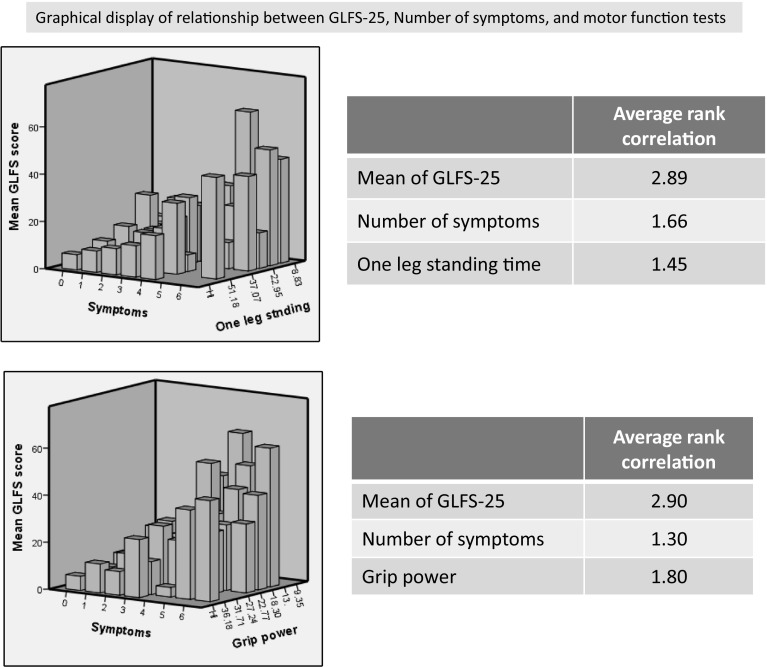
Example relationship among the GLFS-25, number of symptoms and motor functional tests (one-leg standing time and grip power in this graph) clearly shows that the more the number of symptoms, the lower the function, and the higher the mean score of GLFS-25 (Friedman analysis of variance test)

We calculated the number of these symptoms for each participant and revealed significant interrelationship with the GLFS-25 scores (Kruskal–Wallis test) (Fig. 1). All 4 motor functional tests were also significantly related to the GLFS-25 score. The more the number of symptoms, the lower the motor function, and the higher the GLFS-25 mean score. (Friedman test) (Fig. 2).

According to these data, the severity of locomotion dysfunction shown by the GLFS-25 was affected by plural signs and symptoms related to locomotor functions and by results of motor functional tests.

### Laboratory Data or Representative X-ray-Related Variables and the GLFS-25 Score

Three laboratory measures (hyaluronic acid, serum vitamin D, and bone mineral density) were not correlated with the GLFS-25 scores. The FTA did not show any significant correlation with the GLFS-25 scores. However, the LSA, which represents spinal deformity due to degenerative changes in the intervertebral disks or the spinal body, was significantly related to the GLFS-25 scores (Kruskal–Wallis test, *p* = 0.029).

### World Trend on Physical Activity

1. Toronto Charter for Physical Activity 2010

At the third International Congress of Physical Activity and Public Health in Toronto, Canada, 2010, the Global Advocacy for Physical Activity (GAPA) of the International Society of Physical Activity and Health harmonized various opinions and released the “Toronto Charter for Physical Activity” [22]. The Toronto Charter for Physical Activity outlines 4 actions based upon 9 guiding principles and is a call for all countries, regions, and communities to strive for greater political and social commitment to support health enhancing physical activity for all.

2. Global Recommendations on Physical Activity for Health. 2010

The World Health Organization (WHO) developed the “Global Recommendations on Physical Activity for Health” with the overall aim of providing national and regional level policy makers with guidance on the dose–response relationship among the frequency, duration, intensity, type, and total amount of physical activity needed for the prevention of non-communicable diseases [23]. The recommendations in this document address 3 age groups: 5–17 years old, 18–64 years old, and 65 years old and above.

3. Series articles on Physical Activity in The Lancet 2012

One of the leading clinical journals, The Lancet, published series articles on physical activity in 2012 [24–27].

Based on the survey of the literature, the effectiveness of therapeutic exercise in lower limb osteoarthritis is currently well established [28–33]. It is also possible to find aquatic exercises for such diseases [34]. The targets of therapeutic exercise have expanded to chronic non-communicable diseases and other orthopedic diseases as well [35, 36].

The effects of exercise have been investigated not only in terms of mobility and physical functioning but also in terms of the quality of life [37–39].

## Discussion

It has been reported that a large number of people suffer from locomotive dysfunction worldwide, and this number is increasing rapidly [40]. This problem of super-aging cannot be ignored. Unfortunately, as the first country to face this problem, Japan has no model to follow for dealing with this burden.

### Clinical Significance of Orthopedic Problems

Several orthopedic problems covered under locomotive syndrome that are mainly related to the aging process include hip and knee osteoarthritis, degenerative spondylitis, osteoporosis, and lumbar canal stenosis.

Individuals with these conditions are candidates for long-term care insurance. In the past, the literatures have been accumulated regarding each musculoskeletal entity. There are many positive systematic reviews or meta-analyses about the effectiveness of therapeutic exercise in people with these conditions [28–39].

However, an important issue to consider is the complicated condition of multiple involvements in older individuals. These people often develop disabilities; however, in the past, these individuals were identified as single disease bearers. They were not treated as people with multiple involvements. We need a comprehensive approach for people with musculoskeletal disorders, who are often not considered good candidates for therapeutic exercise. Is it still applicable to the people who are not suitable to do exercise due to musculoskeletal diseases?

### Operational Definition and Quantitative Assessment of Locomotor Dysfunction

Operational definition of locomotive syndrome is necessary to study disablement process and to develop intervention strategy for people with disorders. According to the analysis on present study data, clinical symptoms on lumbar spine and lower limbs including pain, ROM and muscle weakness of lower limbs, motor function tests (one-leg standing time, leg extension power, grip power, etc.), and GLFS-25 are necessary to identify people with locomotive syndrome.

The results of the present study clearly showed that the GFLS-25 directly and indirectly reflects diseases, comorbidity, mood, locomotor function, and pain. This can be used as diagnostic criteria as well. According to these data, the severity of locomotive dysfunction in patients sufficiently reflects their multiple orthopedic disorders [6–9]. The GLFS-25 was originally developed as a screening tool for the elderly with locomotive dysfunction. Combined use of this scale with other motor function tests was proven useful [41–43].

From the viewpoint of health promotion corresponding each generation, including; less than 18 years old, 18–65 years old, and more than 65 years old, there are many guidelines or recommendations that suggest physical activity or exercise to be the key factor for maintaining or even promoting health. Such a statement could be valid even in elderly individuals.

Currently, we have not found an effective preventative method to decrease the number of individuals eligible for long-term care insurance. This is because we have studied the effectiveness of exercise for each orthopedic disease as a separate entity. We should conduct studies that assess the comprehensive health status and prevent its decline caused by aging or decreased physical activity [44, 45].

As our knowledge is still limited, we have to connect the both accumulated knowledge.

### Correlation to Geriatric Frailty

The term “geriatric frailty” has been widely used [46, 47]; it covers a wide range of functions in the elderly and includes locomotor function. Similar to locomotive syndrome, frailty is defined operationally and is measured [48–53]. The concept of locomotive syndrome should be evaluated and discussed in comparison with another concept of geriatric frailty.

An important working hypothesis is whether a non-specific approach to maintain or increase physical activity prevents a future care-need condition. Regarding future research, we should investigate the effectiveness of exercise using increased physical activity for selected participants extracted as a group of those with declining locomotive function as evaluated by the GLFS-25.

The indication for increased physical activity should be assessed in those with plural involvements of locomotor function, which might have a negative effect on the effectiveness of exercise.

### Additional Problems and Adherence to Exercise

There is extensive literature supporting therapeutic exercise as useful for maintaining or improving physical activity and for preventing falls. However, important issues need to be resolved; these are as follows: (1) What type of exercise is appropriate in the elderly with knee or back pain and with a deformity of the trunk or lower extremity? (2) What should be the recommended exercise dosage with no adverse effects? (3) How can we measure the effectiveness of interventions?

Another important issue to be investigated is the adherence to exercise. Bauman et al. [54] discussed the reasons for some individuals are being physically active and others being inactive. We ought to study the reasons for some patients being unable to adhere to exercise even though they recognize the importance of such management.

### The Limitation of the Present Study

One of the study limitations was the lack of a longitudinal follow-up. Adequately judging the presence or absence of declining locomotive function in participants and determining the future need for nursing care takes several years. Recognition of changes by repeating the same measures over a long time in the same individuals is important.

Additionally, participants of a prospective cohort study are relatively active and can attend outpatient clinics regularly.

Confirming the efficacy of medical interventions in decreasing the number of older individuals identified by the GLFS-25 is likely to prove difficult, given the time and cost such an investigation would necessitate. Ideally, the prognostic value of the measurement needs to be assessed in prospective studies that investigate patient outcomes with different interventions.

## Conclusion

We proposed and established operational definition for a supposed clinical entity of locomotive organ dysfunction in elderly. Locomotive syndrome is a complex musculoskeletal dysfunction that is assessed based on ADL decline. The GLFS-25 is a comprehensive assessment scale that covers a wide range of ADLs, and it has been proven reliable and valid.

It is important to clarify the mechanism and progression of locomotor system deterioration to prevent care-needed conditions. In the future, we should accumulate evidence of utility of exercise to prevent or delay the deterioration of motor function. For that purpose, we should confirm the feasibility of intervention in those with locomotor dysfunction by musculoskeletal disorders using well-planned therapeutic exercise.
